# Association of TGF-β1 with periodontitis patients in north Indian population

**DOI:** 10.1016/j.jobcr.2025.08.021

**Published:** 2025-08-19

**Authors:** Ruchi Pandey, Sumit Bhateja, Lipika Gopal, Ankita Chhabrani, Anju Rana, Puneet Batra

**Affiliations:** a Department of Periodontology, Manav Rachna Dental College, SDS, MRIIRS, Faridabad, Haryana, 121004, India; b Department of Oral Medicine and Radiology, Manav Rachna Dental College, SDS, MRIIRS, Faridabad, Haryana, 121004, India; c Department of Orthodontics and Dentofacial Orthopedics, Manav Rachna Dental College, SDS, MRIIRS, Faridabad, Haryana, 121004, India

**Keywords:** Periodontitis, Gene polymorphism, North Indian population, TGF-β1 29C/T, -788 C/T

## Abstract

**Introduction:**

Periodontitis is a complex disease caused by environmental and genetic factors. The course of periodontal destruction is influenced by local, ecological, and, predominantly, by genetic factors. Therefore, early detection of the disease through genomics and its course of destruction can be altered through genetic engineering, which can be a major breakthrough in the eradication of periodontitis.

**Aim:**

The purpose of this study is to find the genetic association of TGF-β1 gene polymorphisms in causing periodontitis in the North Indian population.

**Materials and methods:**

A total of 364 subjects were selected in the present study, and they were divided into two groups, viz. Healthy (Group A) and Periodontitis (Group B), with 182 subjects in each. The detailed case history was recorded, buccal swab samples were collected and stored in the sterile medium at 4 °C. Correlation studies on gene polymorphism with the disease occurrence were carried out by estimating allele frequency, Hardy-Weinberg equilibrium (HWE), and chi-square test (GenAlex package), and linkage disequilibrium and haplotype analysis (SHEsis).

**Result:**

The gene TGF-β1 showed a high rate of polymorphism in the north Indian population. The TGF-β1 gene showed polymorphism in the studied SNP sites. Among them, −29C/T and −788 C/T could differentiate the healthy and generalized periodontitis group; however, the localized periodontitis group could not be differentiated with any of the SNP loci.

**Conclusion:**

The gene TGF-β1 showed association with periodontitis in the North Indian Population.

## 1 Introduction

Inevitable plaque deposition, if left unattended, leads to inflammatory changes in the gingival and periodontal tissues, resulting in periodontal breakdown. Periodontitis has a multifactorial origin with three major factors i.e. genetic, environmental, and microbial.^1^

There is a strong correlation between host genetic factors and periodontitis. The research on twins has shown that genetic makeup can change the course of the disease in approximately 50 % of individuals.^2^^,^^3^

It is a known fact that biofilm induces inflammatory responses in the tissues, but the polymorphism may involve single loci, i.e. Single nucleotide polymorphisms (SNPs), or multiple sites may alter the production of inflammatory mediators such as cytokines. Hence, a long inflammatory phase can be observed, resulting in the increased destruction of the supporting structures of the periodontium.

The occurrence of periodontal disease among the Indian population stands at 51 %, with mild to moderate periodontitis accounting for 26.2 % and severe periodontitis comprising 19 %. Females had a lower prevalence compared to males.^4^ Some researchers have focused on the effects of SNPs as candidate genes in causing chronic periodontitis.^5^, ^6^, ^7^, ^8^, ^9^

Approximately 20 genes are found to enhance the risk of having periodontitis; however, ethnicity and factors influenced by the environment play a role in susceptibility to the disease.^10^

One of the cytokines that regulates the course of the periodontal disease is TGF-β1, which is multifunctional and governs cell proliferation, differentiation, death, angiogenesis, and immune reactions. It is also secreted predominantly by macrophages after an injury and controls fibroblasts in synthesizing connective tissues and matrix proteins.^9^^,^^11^

TGF-β exists in three forms. TGF-β1, TGF-β2 and TGF-β3. The various functions of TGF-β, as discussed, are the repair of lesions by activating fibroblastic differentiation and the eradication of inflammation through programmed cell death.^9^

It is a potential marker since levels of TGF-β1 mRNA are expressed more in gingival tissues and are associated with periodontitis.^12^^,^^13^ TGF-β1 gene is located at locus 19q13, polymorphisms at −29C/T,-509 C/T, and −788C/T locations are predominant SNPs associated with periodontal destruction.

The studies mentioned in Table 1 show a well-built relation between TGF-β1 at −509 C/T and periodontitis in the Asian population.

**Table 1:** Summary of TGF-β1 polymorphisms related to periodontitis (Source Self).

Author/Year	Position of Genetic Polymorphism/Race	Associated/Non-associated with Periodontitis
Holla et al., /2002^14^	−988 (C/A), −800 (G/A) and −509 (C/T); other two located at codons 10 (L10P) and 25 (R25P)	Not associated
De Souza et al.,/2003^15^	−509 C/T/Caucasians	Associated
Arab et al., /2012^16^	−509 C/T/Iranian	Associated
Heidari et al., 2013^5^	29 C/T − 509 C/T,788 C/T/Iranian	29 C/T associated
Huang et al./2015^17^	−509C/T (rs1800469), +869T/C (rs1800470), +915G/C (rs1800471)	−509C/T Association with Asians
Cui et al., /2015 Meta-analysis^18^	−509 C/T	Associated
Brodzikowska et al., 2022 meta-analysis^19^	−509C/T T +915G/C CC	Associated in AsiansMay be responsible in Caucasians
Liu et al.,/2022 meta-analysis^20^	−509 C/T	Associated

India shows diversity in the population, periodontitis is multifactorial, and has a high prevalence rate; therefore, it is important to identify specific polymorphisms associated with periodontitis, aiding in early identification and prevention in susceptible patients. The study aims to assess the genetic association between TGF-β1 (−509C/T) polymorphism and periodontitis patients in the North Indian population.

Materials and Methods: A sample size of 364 subjects was segregated into two groups. Group A included 182 systemically and periodontally healthy subjects, and Group B included 182 systemically healthy periodontitis patients. The sample size was estimated through the G-power software (Version 3.1.9.4).

A minimally clinically significant difference for both the markers, that is, TGF-β1 (p1-p2 = 11.2 %)^21^ was found.^15^^,^^21^ A lower minimum clinically significant difference was considered for adequacy of the sample size for both markers. A level of significance (Type 1 error) of 5 %, and power of 80 % (1-Type 2 error) were used for sample size calculation.

The systemically healthy subjects, aged above 18 years (i.e., adult population), with a Gingival Bleeding index (GBI) ≤10 %, no Clinical attachment loss (CAL),^22^ Pocket Probing depth (PPD) ≤ 3 mm,^22^ and Plaque scores ≤1 were included.^23^ For Group B (Periodontitis subjects), subjects with GBI >10 %, CAL 1–4 mm, PPD ≤ 5 mm.^22^ and Plaque scores>1 were included in the study.^23^ Pregnant and lactating females, any form of tobacco users, subjects with known systemic diseases/syndromes or on systemic medication for the past 3 months, and periodontal therapy past six months, with keratosis/pre-cancerous lesions in the buccal mucosa, loss of the cementoenamel junction, and nationality other than Indian (North Indian) were excluded. Study included subjects from the Northern region like Punjab, Uttar Pradesh, Jammu and Kashmir, Uttrakhand, Rajasthan, Gujarat, Delhi, Haryana and Himanchal Pradesh.

Patients visiting the OPD of Manav Rachna Dental College, SDS, MRIIRS were included. The current research got approval from the Institutional Ethical Committee (IEC) MRDC/IEC/PhD/2021/04. Registered with the clinical trial registry as CTRI/2022/09/045992.

**Fig. 1:**
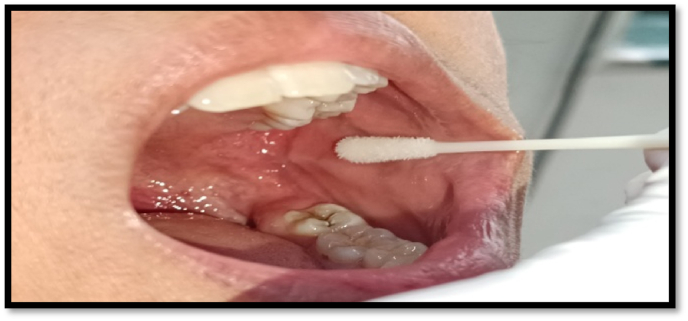
Buccal swab.

The subjects were asked to rinse their mouth gently before collecting the buccal epithelial cells for DNA isolation. The sterile swab was rubbed gently on both buccal mucosa in a back-and-forth motion for approximately 1 min. The collected samples were stored by breaking the swab stick and placing it upside down in a test tube containing DNA/RNA shield. The tube was tightly capped to prevent contamination and stored at 4 °C (Fig. 1).^24^

## 2 Method for DNA analysis

Genomic DNA was segregated from the buccal swab using a Zymo quick DNA mini prep kit, following the guidelines as per the manufacturer, with minor modifications. Purification and quantification of DNA were done using electrophoresis and quantification with a NanoDrop spectrophotometer. Tetra-primer Amplification Refractory Mutation System Polymerase Chain Reaction (T-ARMS PCR) designed by Heidari et al. (2013) was used to amplify and identify the single nucleotide polymorphisms at −509 C/T, 29 C/T, and 788 C/T (Table 2).^5^

**Table 2:** Primers used for TGF-β1 sequencing (Source Self).

Primer Name	Sequencing Primers
TGF β1F	ACACCAACTATTGCTTCAG
TGF β1R	TGTCCAGGCTCCAAATG
TGF β2F	AATTCCTCGAGATAGGCCGT
TGF β2R	TGCGGTTGTGGCAGATATAG
TGF β3F	AACAACATCAACCACAACACAG
TGF β3R	CCGTCTTCCGCTCCTCAG

## 3 Data management and statistical analysis

Demographic and clinical data were tabulated using Microsoft Excel. Allele frequencies and AMOVA were determined using the GenALEx software. SHEsis was used for analyses of linkage disequilibrium**.**

## 4 Sequence quality control and analysis

Priming, trimming, editing, and alignment were performed using Bio Edit software. The ends of the linked sequences were trimmed to minimize the number of missed positions across taxa. Consensus sequences produced after editing were aligned. The MEGA X software was used to test the best-fit nucleotide substitution models. In addition, pairwise distances between the sequences, alignment, and phylogenetic analyses were performed using the MEGA X software.

Analysis of molecular variance (AMOVA) was employed as a statistical tool that made it possible to estimate commonly used F-statistics and/or their analogues and to partition genetic variation among populations and regions hierarchically.

The TGF β1 genotypic frequencies, variant allele carriage, and allelic frequencies for each group were determined using a chi-square (χ2) test. The allelic frequencies were evaluated based on the number of genotypes observed. To validate the distribution of genotypic frequencies, a Hardy–Weinberg equilibrium test was conducted utilizing the χ2 critical value. The odds ratio (OR) was calculated with a 95 % confidence interval (95 % CI), with a p-value of less than 5 % deemed statistically significant. SHEsis, a web-based tool for assessing linkage disequilibrium (LD) among markers and haplotype distributions (http://analysis.bio-x.cn), was utilized.

## 5 Results and Discussion

The total sample size was 364, including 152 males and 212 females. The selected age groups were between 18 and 29 years (247 samples) and above 30 years (117 samples). In the periodontitis group, 101 generalized and 81 localized periodontitis cases were observed (Table 3).

**Table 3:** Demographic Data of the study (Source Self).

	TOTAL SAMPLES (N)	364
**1**	**AGE WISE (years)**	
	≤29	138
	≥30	226
**2**	**GENDER**	
	Male	152
	Female	212
**3**	**DIAGNOSIS**	
	Healthy	182
	Generalized Periodontitis	101
	Localized Periodontitis	81

The frequency of T allele at −509 C/T is higher in the healthy group compared to the C allele frequency present only in the periodontitis group. SNP loci −29 C/T ‘C’ allele was found only in the periodontitis group. However, the 788 C/T 'C’ allele has a higher frequency in the healthy group than in the periodontitis (localized) group. The allele T was only present in the periodontitis group (Table 4). The allele frequency of polymorphic SNP could not differentiate between healthy and periodontitis groups within the gender class and the age groups. However, the SNP loci −29 C/T and −788 C/T could differentiate between healthy and generalized periodontitis groups, as one of the alleles was completely absent from the other. However, the localized periodontitis group could not be differentiated from any other group.

**Table 4:** Genotype and allele polymorphism of TGF-β1 (Source Self).

Polymorphism	Healthy	Generalized Periodontitis	Localized Periodontitis
−509 C/T	182	101	81
C	0.000	0.644	0.778
T	1.000	0.356	0.222
29 C/T	182	101	81
C	0.000	1.000	0.500
T	1.000	0.000	0.500
788 C/T	182	101	81
C	1.000	0.000	0.321
T	0.000	1.000	0.679

Among the haplotypes, the CCT and TCT showed higher genotype frequencies compared to others in differentiating healthy and periodontitis individuals. However, CCT was more significant than the other haplotypes (Table 5).

**Table 5:** Haplotype analysis of TGF-β1 in the healthy and periodontitis populations.

Haplotype	Periodontitis(freq)	Healthy(freq)	Chi^2^	Fisher's p	Pearson's p
T T C∗	24.43(6.7)	364.00(1.000)	636.419	9.99E-16	0.00E+00
C C T∗	199.43 (54.7)	0.00(0.000)	274.68	4.44E-16	0.00E+00
C T C∗	27.57(7.6)	0.00(0.000)	28.653	9.05E-08	0.00E+00
C T T∗	29.00(8.0)	0.00(0.000)	30.203	4.08E-08	3.98E-08
T C T∗	83.57 (23)	0.00(0.000)	94.405	1.89E-15	2.74E-22

**Fig. 2:**
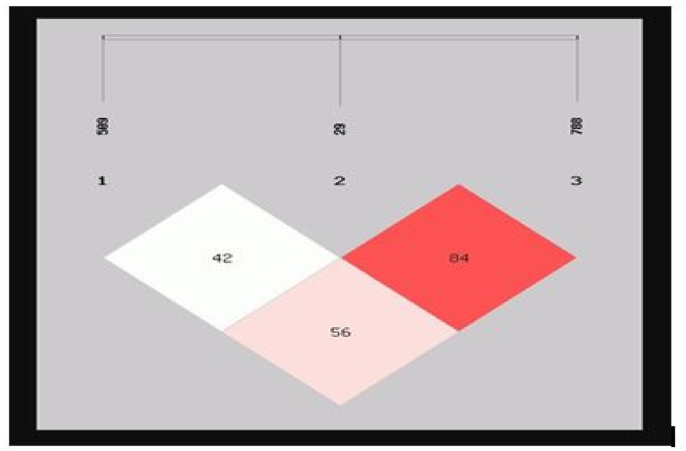
Linkage disequilibrium (LD) pattern of TGF β1 genetic variants in the periodontitis and healthy group (Source Self).

The linkage map showed, −509 and −29 SNPs were predominant in 42 healthy individuals, whereas the combination of −29 and −788 SNPs was observed in 84 subjects. Additionally, co-occurrence of −509 and −788 SNPs was noted in 56 subjects. These findings indicate a higher prevalence of the −29 and −788 SNPs in individuals with healthy periodontium. However, the polymorphism that exists between SNPs would be useful in delineating healthy and generalized periodontitis (Fig. 2).

The analysis of molecular variation also displayed a higher number of variations (71 %) between the populations (healthy vs. localized vs. generalized periodontitis), which could be a marker effective in differentiating healthy subjects from those with periodontitis (Table 6).

**Table 6:** Analysis of molecular variation based on diagnosis (Source Self).

Source	Degree of freedom	Sum of Squares	Mean Square	Estimated Variance.	%
**Among Populations**	2	367.106	183.553	0.808	71 %
**Among Individuals**	361	29.645	0.082	0.000	0 %
**Within Individuals**	364	120.500	0.331	0.331	29 %
**Total**	727	517.251		1.139	100 %

TGF-β1 is a multifaceted cytokine that controls a wide variety of biological processes, including angiogenesis, cell division, apoptosis, and immune reactions.^11^ It has been found to involve a predominant role in the synthesis of connective tissue, fibronectin, periodontal ligament cells, proteoglycans, glycosaminoglycans, and many types of cells. TGF-β1 is abundant and pervasive compared with its homologous form. Genetic risk factors may differ among populations; therefore, it is important to determine risk factors in every population. Thus, the current study results showed that TGF-β1-509 C/T is more prevalent in the healthy group compared to the C allele frequency present only in the periodontitis group.

In the present study, the TGF-β1 SNP at loci 29 C/T and C alleles was found only in the periodontitis group. Whereas, in 788 C/T, the C allele was found more frequently in the healthy group than in the periodontitis (localized) group, and the T allele was only present in the periodontitis group. The allele frequency of polymorphic SNP could not differentiate between the healthy and periodontitis groups within the gender and age groups. However, the SNP loci 29 C/T and 788 C/T could differentiate between healthy and generalized periodontitis groups, as one of the alleles was completely absent in the other. However, the localized periodontitis group could not be differentiated from any other group. To compare the present study results, there is no evidence in the literature pertaining to similar studies on the Indian population. This elemental study was conducted in Indians to find an association between TGF-β1 and periodontitis. However, studies conducted globally have been critically evaluated in order to compare our results.

Cui et al. (2015) studied the link between TGF-β1 rs1800469 polymorphism and the prevalence of periodontitis.^18^

Meta-analysis of immunomodulatory gene polymorphisms by Heidari et al. (2019) concluded that the TGF β1 codon 25 can increase the risk of periodontal disease and found a strong link between the TGF-β1-509 SNP and periodontitis in Asians.^25^

A similar study by Hassan et al. (2019) found an association of chronic periodontitis and TGFβ1-509 C/T in 75 subjects.^26^

A Romanian study by Nica et al. (2019) identified the varied distribution of TGF β1 C-509T and IL-6 G-174C polymorphisms in diseased and healthy groups. Nonetheless, no correlation was found between the occurrence of TGF β1 C-509T variants and the likelihood of periodontitis in males. Studies conducted by other researchers worldwide on TGF-β1 and its association with periodontitis have shown conflicting results. The present study results are in accordance with the studies mentioned above.^27^

Our study results are in sync with the above-mentioned studies of TGF β1 but may be at different loci, which can be attributed to different regions, diets, environmental conditions, and their genetic makeup.

## 6 Conclusion

The TGF-β1 gene showed a high rate of polymorphism in the North Indian population. Linkage analysis showed the TGF-β1 gene showed polymorphism at the studied SNP sites. Among them, −29C/T and −788 C/T could differentiate between the healthy and generalized periodontitis groups; however, the localized periodontitis group could not be differentiated from any SNP loci.

### 6.1 Limitations of the study

Analysis of different variables, including different classes of age groups, smokers, and non-smokers, was not done. Additionally, due to limited sample size and insufficient representation across different stages of periodontitis, it was not possible to establish a clear association between SNP loci of the studied genes and the severity or progression of the disease.

## Source of funding

This is a self funded PhD work of Dr Ruchi Pandey.

## Declaration of competing interest

The authors declare the following financial interests/personal relationships which may be considered as potential competing interests: Reports a relationship with that includes:. Has patent pending to. If there are other authors, they declare that they have no known competing financial interests or personal relationships that could have appeared to influence the work reported in this paper.
